# KL-6 as a Biomarker for Adult Patients with Cystic Fibrosis and the Impact of *MUC1* Genotype

**DOI:** 10.3390/jcm15124555

**Published:** 2026-06-12

**Authors:** Sarah Ricken, Sarah Dietz-Terjung, Gerhard Weinreich, Jose Ortiz, Michaela Schedel, Svenja Straßburg, Christian Taube, Matthias Welsner, Francesco Bonella, Sivagurunathan Sutharsan

**Affiliations:** 1 Department of Pulmonary Medicine, University Medical Center Essen-Ruhrlandklinik, 45239 Essen, Germany; sarah.ricken@rbk.de (S.R.); sarah.terjung@uk-essen.de (S.D.-T.); michaela.schedel@rlk.uk-essen.de (M.S.); 2 Department of Pulmonary Medicine, University Medical Center Essen, University of Duisburg-Essen, 45141 Essen, Germany; 3 Department of Sleep and Telemedicine, University Medical Center Essen-Ruhrlandklinik, University of Duisburg-Essen, 45239 Essen, Germany; joseguillermo.ortizsucre@rlk.uk-essen.de; 4 Department of Pulmonary Medicine, University Medical Center Essen-Ruhrlandklinik, Adult Cystic Fibrosis Center, University of Duisburg-Essen, 45239 Essen, Germany; gerhard.weinreich@rlk.uk-essen.de (G.W.); svenja.strassburg@rlk.uk-essen.de (S.S.); christian.taube@rlk.uk-essen.de (C.T.);; 5 Department of Pulmonary Medicine, Ruhrlandklinik, University Medicine Essen, German Center for Lung Research (DZL), University of Duisburg-Essen, 45239 Essen, Germany; 6 Center for Interstitial and Rare Lung Diseases, Pneumology Department, University Medical Center Essen-Ruhrlandklinik, 45239 Essen, Germany

**Keywords:** KL-6, cystic fibrosis, biomarker, *MUC1*, single nucleotide polymorphism, disease progression, exacerbation, personalized medicine

## Abstract

**Background/Objectives**: Krebs von den Lungen-6 (KL-6) is a mucin-like glycoprotein that is elevated in a variety of lung diseases and used as a diagnostic and prognostic biomarker in people with cystic fibrosis (pwCF). Single nucleotide polymorphisms (SNPs) in *mucin-1* (*MUC1*) influence KL-6 serum concentration. This study investigated the relationship between serum KL-6 concentrations in pwCF and a *MUC1* SNP and its longitudinal dynamics. **Methods**: The study included pwCF (*n* = 174) and healthy controls (*n* = 30). In pwCF, 365 samples were collected for longitudinal analyses; KL-6 levels were measured and the *MUC1* SNP rs4072037 was genotyped in pwCF and controls. Cross-sectional and longitudinal associations between KL-6, genotype, and clinical parameters, such as infectious exacerbation, body mass index, inflammatory values and lung function, were analyzed using linear mixed-effects models. **Results**: Serum KL-6 was significantly elevated in pwCF compared with controls (458 ± 357 vs. 283 ± 103 U/mL; *p* < 0.001). Homozygous G/G carriers exhibited higher baseline KL-6 than A/A carriers (627 ± 673 vs. 397 ± 148 U/mL; *p* < 0.001), while heterozygous individuals showed intermediate levels. Longitudinally, the *MUC1* SNP and interindividual differences in vital capacity (ppFVC) primarily determined baseline KL-6 levels, explaining 52.5% of variance. Short-term intraindividual fluctuations were largely driven by infectious exacerbations independent of genotype, accounting for ~10% of within-subject variance. **Conclusions**: PwCF generally showed elevated serum KL-6 levels and reflected both stable interindividual differences, mainly driven by the *MUC1* SNP and ppFVC. Dynamic intraindividualchanges were associated with infectious exacerbations. Given the influence of *MUC1* polymorphisms (e.g., rs4072037) on KL-6 concentration, personalized interpretation based on the genotype status may be informative in pwCF.

## 1. Introduction

Cystic fibrosis (CF) is an autosomal recessive genetic disorder caused by mutations in the *cystic fibrosis transmembrane conductor regulator* (*CFTR*) gene, affecting over 100,000 individuals worldwide and leading to a multisystem disease with significant pulmonary involvement [1,2,3]. These mutations result in decreased quantity and/or function of the CFTR protein, an anion channel expressed on epithelial cells in multiple organs [1,3,4]. The disease manifests with symptoms such as chronic cough, obstructive bronchitis, recurrent pneumonia, and eventually respiratory insufficiency due to progressive lung destruction [5]. Comorbidities frequently include chronic sinusitis, pancreatic insufficiency, liver disease, osteoporosis, and gastrointestinal complications. Psychological challenges such as anxiety and depression are also common [6].

Therapeutic strategies include inhaled mucolytics, antibiotics, and physiotherapy. CFTR modulators such as ivacaftor (a potentiator) and correctors like lumacaftor, tezacaftor, vanzacaftor and elexacaftor aim to restore CFTR protein function, particularly in individuals carrying the most common *F508del* mutation [7,8,9,10]. In advanced stages of disease, lung transplantation may be required, while palliative care, including non-invasive ventilation, remains an option when transplantation is not feasible [1].

KL-6, encoded by the *mucin-1* (*MUC1*) gene, is expressed on type II alveolar epithelial cells and is elevated in a range of pulmonary diseases, including interstitial lung diseases [11,12], non-small cell lung cancer [13], and CF [14]. KL-6 can be detected in serum and bronchoalveolar lavage fluid and has gained attention as a diagnostic and prognostic biomarker in conditions such as pleural mesothelioma [15], diffuse alveolar hemorrhage [16], and SARS-CoV-2 pneumonia [17]. It has also been shown to predict acute exacerbations in idiopathic pulmonary fibrosis [18], chronic lung allograft dysfunction in lung transplant recipients [19], and disease severity and clinical outcomes alveolar proteinosis [13].

As a predictive biomarker, elevated KL-6 levels are associated with pulmonary disease progression and an increased risk of exacerbations [12,18]. In patients with CF (pwCF), we previously demonstrated that serum KL-6 levels were significantly elevated compared with age- and sex-matched healthy controls and correlated inversely with lung function parameters, including percent predicted forced expiratory volume in one second (ppFEV_1_) and percent predicted forced vital capacity (ppFVC) [14]. KL-6 showed superior diagnostic performance compared with conventional inflammatory markers such as C-reactive protein (CRP) and lactate dehydrogenase (LDH), while KL-6 concentrations were independent of pancreatic insufficiency, chronic infection status, hospitalization frequency, sex, age, and inhaled or oral therapies [14].

As a preventive measure, routine KL-6 monitoring may allow for early detection of epithelial injury or subclinical exacerbation, enabling proactive interventions and reducing long-term lung damage [17,18]. For different diseases including pulmonary alveolar proteinosis [13], interstitial lung diseases [20,21,22,23], and antisynthetase syndrome-associated interstitial lung disease [24], the KL-6 serum concentration is influenced by genetic variants in the *MUC1* gene, particularly by the single nucleotide polymorphism (SNP) rs4072037, located in an exonic region leading to an amino acid change; individuals homozygous for the polymorphic allele (G/G) have been reported to show higher KL-6 levels than those with A/G or A/A genotypes, suggesting allele-specific reference values [13,25].

Our study aims to assess KL-6 levels in relation to the genotype status of rs4072037 in pwCF compared with healthy controls and to evaluate its potential as a biomarker for monitoring disease severity, predicting outcomes, enabling personalized care, and supporting participatory medicine in CF.

## 2. Materials and Methods

### 2.1. Patient Population, Clinical Data, and Clinical Definitions

PwCF, who received treatment at the Ruhrlandklinik Essen, Germany, between 12 March 2015 and 12 January 2018, were eligible for inclusion in the study. CF diagnosis was confirmed by evidence of CFTR dysfunction, either through pathological chloride sweat test results in at least two independent measurements or by the presence of two CF-causing *CFTR* mutations. The control group consisted of healthy, non-smoking individuals without any known pulmonary disease. Control samples were provided by Bonella et al. and originated from a previously conducted, ethically approved study [13]. All participants provided informed consent for the use of blood and serum as well as associated clinical data. The West German Biobank Essen (WBE) approved the protocol, including permission for genomic analyses, under the approval of the local ethics committee (ethics approval no. 17-7667-BO). The study protocol approved by the local ethics committee (approval no. 17-7667-BO) explicitly included the analysis of biobanked samples as well as the prospective collection of additional samples and clinical follow-up data.

Accordingly, the present investigation comprised a retrospective analysis of previously stored biobank samples and associated clinical data together with prospectively collected samples obtained after ethics approval within the same approved observational protocol. No study-specific visits, blood sampling procedures, or additional patient appointments were performed for the purpose of this study. All biological samples and clinical data were obtained during routine outpatient follow-up visits or hospitalizations as part of standard clinical care and were analyzed retrospectively.

The data included demographic parameters (age, sex, body mass index (BMI)), *CFTR* genotype, sweat chloride concentration, lung function, pancreatic function, inflammatory markers (C-reactive protein (CRP), blood leukocytes, serum IgG (IgG)), CF-related diabetes, infectious exacerbations, and microbial colonization of sputum. For longitudinal analyses, all available serum samples from 174 pwCF were included, resulting in 365 samples in total (1–5 samples per patient, mean interval 5.68 months). Patients were categorized based on their *CFTR* mutation and chronic pathogen colonization. Pancreatic insufficiency was defined by the need for enzyme substitution, and diabetes mellitus was defined according to specific guidelines [26].

An infectious exacerbation was defined by the presence of at least one of the following criteria: (i) an increase in CRP levels of >2 mg/dL compared with the patient’s previous value, (ii) initiation of antibiotic therapy or modification of an ongoing antibiotic regimen, or (iii) clinical deterioration characterized by a combination of changes in sputum color, increased sputum production, enhanced expectoration, and/or fever.

### 2.2. KL-6 Quantification

KL-6 concentration was measured in serum samples from 174 pwCF, from whom a total of 365 serum samples were collected longitudinally, and 30 healthy controls using the Nanopia^®^ KL-6 Reagent (Sekisui Medical Co., Ltd., Tokyo, Japan), as previously described [13]. The first blood sample obtained from each individual was defined as the baseline KL-6 level. Briefly, 150 µL of buffer solution (Reagent 1) was added to 2.5 µL of serum. After a 5 min incubation, Reagent 2 containing the monoclonal KL-6 antibody was added. Changes in absorption at 570/800 nm after 5 min were measured using the ADVIA^®^ 1800 Clinical Chemistry System (Siemens Healthineers AG, Erlangen, Germany), calibrated with the Nanopia^®^ KL-6 Calibrator (Sekisui Medical Co., Ltd., Tokyo, Japan).

### 2.3. Genotyping

DNA was isolated from serum samples stored in the WBE by the WBE using standard procedures for serum DNA extraction. The SNP rs4072037 was genotyped in 174 pwCF and 30 healthy controls using the TaqMan^®^ SNP Genotyping Assay (Thermo Fisher Scientific (Applied Biosystems), Waltham, MA, USA), based on real-time polymerase chain reaction with fluorescence measurements. The assay included primers and TaqMan^®^ MGB probes to detect different alleles. The probes contained a reporter dye (VIC^®^ or FAM™) at the 5′ end and a quencher dye at the 3′ end. Each sample was measured in duplicate as a technical replicate, and samples yielding an undetermined result in either replicate were excluded, resulting in a final genotyped cohort of 158 pwCF.

### 2.4. Statistical Analyses

For descriptive analyses, both absolute numbers and percentages were used for categorical data, as well as mean and standard deviation for continuous data. Patients were categorized into subgroups based on their *MUC1* SNP (homozygous for the wildtype allele: A/A, heterozygous A/G, or homozygous for the polymorphic allele: G/G). The analysis initially used one measurement per participant to ensure sample independence. The Kolmogorov–Smirnov test was used to test normality of distribution. Between-group comparisons of non-normally distributed variables were analyzed using the Mann–Whitney U test or Kruskal–Wallis test, with post hoc Dunn–Bonferroni tests. Associations between KL-6 concentration and continuous variables were assessed using Spearman’s rank correlation. Effect sizes were calculated according to Cohen’s formula. Results with a *p*-value < 0.05 were considered statistically significant.

Longitudinal analyses were conducted using linear mixed-effects models (LMMs) to account for repeated measurements within individuals and to model both intra- and interindividual variability. KL-6 concentrations were log-transformed to meet model assumptions. Fixed effects included *MUC1* genotype, lung function (FEV1, VC), inflammatory markers [CRP, leukocytes, immunoglobulin G (IgG)], BMI, and pulmonary exacerbations. Continuous covariates were decomposed into between-person (interindividual) and within-person (intraindividual) components by centering around the individual mean, allowing separate estimation of associations driven by differences between patients versus temporal changes within patients. Random effects at the patient level were initially included but removed when found redundant. The significance of fixed effects was evaluated using F-statistics derived from the LMMs, with degrees of freedom estimated according to the Satterthwaite approximation. Estimated marginal means (EMMs) were calculated and back-transformed to the original scale (U/mL) for interpretation. *p*-values were derived from model-based fixed-effect estimates, and no correction for multiple testing was applied. Due to the small number of heterozygous carriers, analyses in this subgroup were considered exploratory.

Data analysis was performed using IBM SPSS Statistics for Windows, Version 29.0 (IBM Corp., Armonk, NY, USA).

## 3. Results

### 3.1. Patient Demographics

A total of 174 pwCF were included (58.6% male; mean age at sample collection: 33 ± 12.0 years; range: 16–71 years) (Table 1), along with 30 healthy control subjects (63.3% male; mean age: 41 ± 10.8 years; range: 20–64 years). Age did not significantly differ between pwCF and healthy controls (41.4 ± 10.8 vs. 32.3 ± 11.6 years; *p* > 0.05), and no significant difference in sex distribution and any other clinical parameter was observed between the groups (*p* > 0.05). On average, 1.7 measurements were collected per patient (range: 1–5), with a mean interval of 5.7 months between the first and subsequent samples (range: 0–24 months). KL-6 values were missing for seven of the 365 samples (1.9%).

### 3.2. MUC1 Polymorphism rs4072037

Genotyping of rs4072037 was successful in 158 pwCF and 30 healthy controls. Minor allele frequencies (MAFs) were calculated for each cohort independently. Comparable to the expected MAF of a cohort of European decent (allele frequency aggregator, MAF = 0.47) [8], a MAF of 0.50 was observed in the healthy control group and genotypes were in Hardy–Weinberg equilibrium (HWE) (χ^2^ = 1.2, *p* = 0.27). In contrast, the MAF was 0.26 in pwCF deviating from HWE as expected due to disease selection (Table 2).

In the control group, the distribution of the *MUC1* polymorphism rs4072037 was that nine were homozygous carriers of the A/A genotype (30.0%), 12 individuals (40%) were heterozygous (A/G) and nine were homozygous for G/G (30.0%) (Table 2). In 158 pwCF, 66.1% were homozygous for the wildtype allele (A/A)), five (2.9%) pwCF were heterozygous and 21.8% were homozygous for the polymorphic allele G/G). Genotyping was unsuccessful in 16 patients (9.2%) due to insufficient DNA quality or concentration.

No genotype-dependent differences were observed in any clinical parameter analyzed in pwCF.

### 3.3. KL-6 Concentrations in pwCF and Association with the MUC1 Genotype of rs4072037

**Figure 1:**
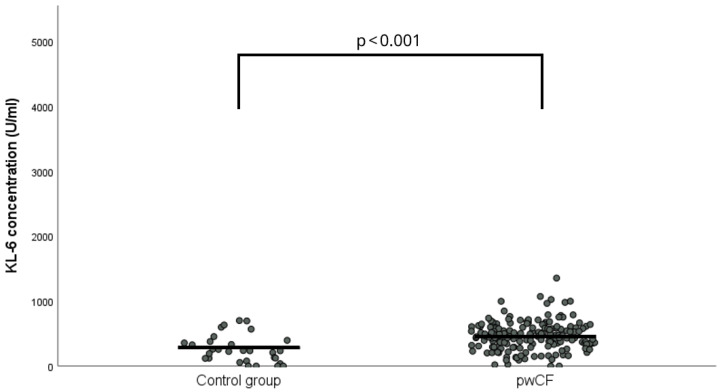
KL-6 concentrations in pwCF (*n* = 174) compared with healthy controls (*n* = 30) at baseline. Mean concentrations are shown as a horizontal line. Statistical comparison was performed using the Mann–Whitney U test.

The mean ± SD KL-6 concentration was significantly higher in pwCF at baseline compared with the control group (458.08 ± 356.97 U/mL vs. 282.93 ± 103.10; *p* < 0.001; effect size 0.33) (Figure 1).

In pwCF, a statistically significant difference in KL-6 concentrations at baseline depending on the *MUC1* genotype of rs4072037 (*p* < 0.05) was observed. Subsequent pairwise comparisons showed no statistically significant difference in KL-6 concentration between homozygous carriers of the A allele and heterozygous pwCF (*p* = 0.302). Similar effects were observed in heterozygous individuals compared with homozygous pwCF of the polymorphic allele (*p* = 1.000). Significantly higher KL-6 levels were associated in pwCF with the homozygous G versus A genotype (*p* = 0.000015) (Table 3).

Additional analyses using dominant and recessive models in pwCF confirmed these findings as KL-6 concentrations were significantly higher in patients carrying at least one G allele, supporting a genotype-dependent effect of the *MUC1* rs4072037 variant on KL-6 levels in pwCF (Table 3).

Although KL-6 levels within the control group were numerically higher in homozygous carriers of the polymorphic allele than in homozygous carriers of the wildtype allele or heterozygous individuals, these differences for the *MUC1* SNP rs4072037 were not statistically significant. Similarly, the dominant or recessive model did not reveal a genotype-dependent effect on KL-6 levels in controls (Table 3).

However, KL-6 levels were significantly higher in pwCF compared with controls across all genotype groups: A/A (median 397 vs. 251 U/mL, Mann–Whitney U = 817, *p* = 0.002), A/G (median 523 vs. 267 U/mL, Mann–Whitney U = 57, *p* = 0.004), and G/G (median 672 vs. 337 U/mL, Mann–Whitney U = 268, *p* = 0.004, exact test).

### 3.4. Baseline Associations Between KL-6 Concentrations and Clinical Characteristics

KL-6 concentrations were significantly associated with several clinical and inflammatory parameters at baseline (Table 4):

A negative correlation was observed between KL-6 concentrations and BMI (Spearman’s ρ = −0.304, *p* < 0.001). In addition, KL-6 levels were significantly higher in pwCF presenting with an infectious exacerbation at baseline compared with those without exacerbation (*p* < 0.001).

Regarding inflammatory markers, KL-6 concentrations showed moderate positive correlations with CRP (Spearman’s ρ = 0.429, *p* < 0.001) and leukocyte counts (Spearman’s ρ = 0.328, *p* < 0.001), as well as a weak but statistically significant positive correlation with IgG levels (Spearman’s ρ = 0.263, *p* = 0.005).

Furthermore, KL-6 concentrations were strongly negatively correlated with lung function parameters, including ppFVC (Spearman’s ρ = −0.610, *p* < 0.001) and ppFEV_1_ (Spearman’s ρ = −0.608, *p* < 0.001).

In contrast, no significant associations were observed between KL-6, age, sex, *CFTR* mutation class, pancreatic insufficiency, or the presence of CF-related diabetes mellitus (CFRD). Likewise, correlations between KL-6 and glycosylated hemoglobin (HbA1c), calprotectin, and sweat chloride concentration were weak and not statistically significant.

KL-6 concentrations tended to be higher in participants colonized with Pseudomonas aeruginosa (582.8 ± 694.5 U/mL, *n* = 34) or multiple pathogens (467.6 ± 183.2 U/mL, *n* = 81) compared with those without colonization (356.3 ± 141.0 U/mL, *n* = 24) or single non-Pseudomonas organisms, although pairwise post hoc comparisons were not significant after Bonferroni correction.

Stratified analyses by the *MUC1* genotype of rs4072037 were performed to assess whether the genotype modified the associations between KL-6 concentrations and BMI, infectious exacerbations, inflammatory markers (CRP, leukocytes, IgG), and lung function. Across genotypes, negative correlations were observed between KL-6 and BMI, as well as KL-6 and lung function (ppFVC and ppFEV_1_), with statistical significance in homozygous carriers of the A and the G allele (Spearman’s ρ ranging from −0.313 to −0.750, all *p* < 0.01, Table 5). Positive correlations between KL-6 and CRP, leukocyte counts, and IgG were also significant for pwCF homozygous for the wildtype and the polymorphic allele (Spearman’s ρ 0.270–0.523, all *p* ≤ 0.01).

The presence of infectious exacerbation was associated with higher KL-6 levels in G/G carriers (*p* = 0.009) but not in patients with at least one A allele. Fisher Z comparisons of correlation coefficients revealed no significant differences between genotypes for any parameter, suggesting that the *MUC1* genotype did not significantly modify these associations (Table 5).

### 3.5. KL-6 Concentrations over Time

Given the significant associations of KL-6 with markers of inflammation, lung function, and nutritional status at baseline, the distribution of these clinical parameters was subsequently examined across all longitudinal samples and stratified by the *MUC1* genotype of rs4072037. Clinical parameters were recorded at the time of each sample collection. Overall, the longitudinal distributions were comparable to baseline characteristics (Table 4). Considering the unequal genotype group sizes and the presence of repeated measurements per individual, no formal between-group statistical comparisons were conducted.

In longitudinal mixed-effects models (LMMs), interindividual effects reflect differences in KL-6 levels between subjects, whereas intraindividual effects represent within-subject changes over time with log-transformed KL-6 as the dependent variable (Table 6). In this LMM, the *MUC1* genotype emerged as the strongest determinant of overall KL-6 levels. Estimated marginal means demonstrated significantly higher KL-6 concentrations in G/G carriers compared with A/A carriers (back-transformed EMMs approximately 542 U/mL vs. 348 U/mL; *p* < 0.001), whereas pwCF heterozygous for rs4072037 did not differ significantly from either group. Thus, the genotype primarily determined the baseline level of KL-6. Among clinical parameters, only interindividual ppFVC was independently associated with KL-6 (*p* < 0.001). No significant effects were observed for BMI, CRP, leukocytes, IgG, ppFEV_1_, or chronic exacerbation status in the overall model. The model explained 52.5% of the variance in KL-6 concentrations (marginal R^2^ = 0.525).

**Figure 2:**
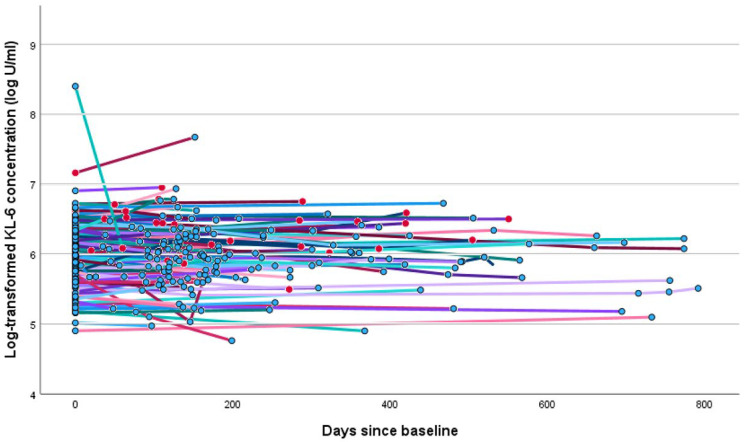
Longitudinal log(KL-6) trajectories (log U/mL) over days since baseline in patients with ≥2 measurements. We used a truncated y-axis to improve visualization of individual trajectories; the full scale does not include zero due to log transformation. Each colored line represents an individual pateint; blue points indicate stable periods, red points indicate infectious exacerbations.

When focusing specifically on intraindividual changes over time, infectious exacerbation was the only significant predictor of KL-6 dynamics (*p* < 0.001) (Figure 2). EMMs showed higher KL-6 levels during exacerbation episodes compared with clinically stable phases (518 U/mL vs. 370 U/mL). The intraindividual model explained approximately 10% of the within-subject variance in KL-6. Time since baseline and all other time-varying clinical parameters were not significant (Table 7).

Genotype-stratified analyses confirmed that exacerbations were associated with significant intraindividual increases in KL-6 in both in pwCF homozygous of the A or the G allele (both *p* < 0.001). Additional associations with BMI and IgG were observed only in pwCF with the homozygous G genotype. However, no significant genotype-by-predictor interactions were detected overall.

Taken together, these findings indicate that the *MUC1* genotype of rs4072037 determines the absolute KL-6 level and interindividual differences in ppFVC contribute to between-patient variability. In addition, infectious exacerbations drive short-term intraindividual fluctuations, which was independent of the *MUC1* genotype.

## 4. Discussion

Our study demonstrates for the first time in pwCF that serum KL-6 concentrations are shaped by both genetic background and acute inflammatory events. The MUC1 rs4072037 genotype was the dominant determinant of baseline KL-6 levels, with homozygous polymorphic carriers showing higher concentrations than wildtype individuals, while interindividual differences in ppFVC further contributed to between-patient variability. Short-term intraindividual fluctuations were primarily driven by infectious exacerbations, independent of the genotype. Estimated marginal means indicated that acute increases during exacerbations explained ~10% of within-subject variance, whereas genotype and ppFVC accounted for ~52% of overall variance. This distinction between genetically determined baseline expression and inflammation-associated dynamics provides a novel perspective on KL-6 as a biomarker in chronic pulmonary disease.

KL-6 is a recognized biomarker for various interstitial and inflammatory lung diseases [11,12,13,15,16,18,19] and has also been linked to infectious processes [17] with emerging relevance in pwCF [14]. Consistent with previous studies, we observed significantly elevated mean KL-6 concentrations in pwCF compared to lung-healthy controls [11,12,14]. An earlier study by Bonella et al. demonstrated elevated serum KL-6 levels in pwCF compared with healthy controls and reported an inverse correlation with lung function parameters [14]. Although a small number of individuals overlapped between that cohort and the present study population, the majority of patients included here represent a distinct and independent cohort. Importantly, our findings confirm the previously reported association between KL-6 and lung function in pwCF and substantially extend these observations by incorporating longitudinal sampling and genotype-stratified analyses. In contrast to the earlier cross-sectional approach, the present study demonstrates that absolute KL-6 concentrations are predominantly determined by *MUC1* genotype, whereas short-term intraindividual fluctuations are driven by infectious exacerbations. This integrated genetic and longitudinal perspective provides a more comprehensive understanding of KL-6 regulation in CF.

We found that a *MUC1* polymorphism significantly influenced KL-6 concentrations in the serum. Individuals with the homozygous polymorphic allele exhibited markedly higher serum KL-6 levels than those with the wildtype genotype. Heterozygous individuals showed numerically intermediate KL-6 concentrations, but differences with either homozygous group were not statistically significant. This genotype-specific response suggests that host genetic factors may modulate epithelial cell turnover, mucin production, or biomarker clearance, contributing to individualized KL-6 profiles in pwCF [20,21,22,23,25]. The lack of statistical significance for the heterozygous pwCF for rs4072037 may be attributable to the low number of samples in this group.

Importantly, our longitudinal analyses revealed that KL-6 dynamics reflect both inter- and intraindividual processes. The *MUC1* genotype of rs4072037 emerged as the strongest determinant of absolute KL-6 levels, with G/G homozygotes exhibiting markedly higher concentrations than A/A carriers. Interindividual differences in ppFVC additionally contributed to between-patient variability. Together, the model explained 52.5% of the overall variance in KL-6 concentrations, highlighting the dominant role of genotype and ppFVC differences among patients. The functional consequences of rs4072037 remain incompletely understood. Studies in pulmonary alveolar proteinosis, interstitial lung disease, and lung adenocarcinoma suggest genotype-dependent differences in *MUC1* expression and KL-6 levels in related contexts [13,20,21,24].

Previous studies investigating the relationship between rs4072037 and KL-6 have largely focused on other pulmonary conditions such as pulmonary alveolar proteinosis [13] and interstitial lung diseases [20,21,22,23]. In antisynthetase syndrome-associated ILD, genotype-dependent differences in serum KL-6 levels have also been described [24]. In these settings, genotype-dependent differences in KL-6 concentrations were primarily interpreted as reflecting disease-specific alterations in alveolar epithelial injury or *MUC1* expression [20,21]. Our study extends these observations to CF, a genetically determined, chronic suppurative lung disease with distinct pathophysiological mechanisms characterized by impaired mucociliary clearance, persistent infection, and neutrophil-dominated inflammation [2,5].

While earlier reports suggested that rs4072037 modulates baseline KL-6 expression in interstitial or malignant lung pathology [20,24], our findings demonstrate that this genetic influence is also evident in pwCF and remains the dominant determinant of absolute KL-6 levels even in the presence of chronic infection and structural lung damage. Importantly, we further show that the genotype status primarily determines interindividual baseline differences, whereas short-term intraindividual fluctuations are driven by infectious exacerbations. This differentiation between genetically determined baseline expression and inflammation-associated dynamics has not been described previously and adds a novel dimension to the interpretation of KL-6 as a biomarker.

Our study not only corroborates genotype-dependent KL-6 variation as observed in other pulmonary diseases [13,20,24], but also contextualizes it within the unique inflammatory and genetic landscape of CF. These findings highlight that KL-6 biology is not disease-specific per se, but may reflect an interaction between constitutive genetic regulation and superimposed epithelial stress [20,25]. Consequently, interpretation of KL-6 concentrations in clinical practice should account for both the underlying genotype and disease context.

In contrast, short-term intraindividual fluctuations in KL-6 were primarily driven by infectious exacerbations and were independent of the *MUC1* SNP under study. Estimated marginal means showed transient increases during exacerbation episodes, with the intraindividual model explaining approximately 10% of within-subject variance. No other time-varying clinical parameters significantly influenced KL-6 dynamics. These findings underscore that KL-6 may be sensitive to acute epithelial stress while remaining largely stable under baseline conditions dictated by the underlying genetic background. In line with the principles of predictive and preventive medicine, serum KL-6 may have potential as an adjunctive biomarker for detecting early epithelial stress before functional decline becomes clinically apparent.

These results emphasize the importance of personalizing KL-6 interpretation in pwCF. Absolute cut-off values may be of limited utility, especially when genetic variation affects baseline concentrations. Instead, monitoring relative intraindividual changes may offer more clinically meaningful insights. Within the framework of precision medicine, KL-6 could thus support individualized therapy management by enabling early detection of physiological changes and guiding therapeutic decision-making [20,24].

KL-6 may help stratify patients according to their risk for functional decline or pulmonary complications and thereby support participatory care models through biomarker-guided monitoring. In this context, the potential value of genotype-based KL-6 levels for assessing the disease course or therapeutic response is of particular interest. However, genotype-specific analyses and the effect of treatment on serum KL-6 levels, including highly effective CFTR modulator therapies such as vanzacaftor/tezacaftor/deutivacaftor and elexacaftor/tezacaftor/ivacaftor, were not addressed in the present study and need to be the subject of future investigations.

Despite these strengths, our study has several limitations. The genotype distribution of rs4072037 deviated from HWE in our cohort of pwCF, with a pronounced heterozygote deficiency. This contrasts with previous studies in other pulmonary and systemic conditions, including pulmonary alveolar proteinosis [13] and ASSD/ILD [24], where HWE conformity was reported. These were typically conducted in balanced 1:1 case–control designs and may more closely reflect general population allele frequencies. While the MAF in our control group corresponded to that expected for European populations, the MAF in pwCF was substantially lower, indicating that the deviation is specific to the CF group rather than a general genotyping artifact. Allele frequency data for rs4072037 in other cohorts of pwCF has previously not been investigated. Given the substantially larger number of pwCF compared to controls in our study, and the disease-specific nature of this cohort, violation of HWE assumptions due to disease-related selection, survival effects, or linkage to CF-relevant genetic backgrounds is plausible. SNP rs4072037 is located in the *MUC1* gene encoding KL-6 and the observed significant association with KL-6 levels, a biologically meaningful effect within the CF population can be assumed. However, these findings require replication in independent CF cohorts to explore the potential underlying mechanisms. In addition, the retrospective design, limited observational period, small subgroups, and potential confounders such as heterogeneous time intervals between measurements and an ethnically non-diverse cohort must be considered when interpreting the results. Nonetheless, these findings support the hypothesis that KL-6 not only reflects pulmonary disease activity but is also influenced by genetic background. This highlights the biomarker’s promise in predictive, preventive, and personalized CF care. Future studies should prospectively validate KL-6 as a longitudinal biomarker and explore its integration into clinical routines across diverse populations.

## 5. Conclusions

This study demonstrates that serum KL-6 concentrations are elevated in pwCF compared with healthy controls and that these levels are influenced by a synonymous *MUC1* SNP. Our findings underscore the dual nature of KL-6 as a biomarker: its baseline levels are largely determined by genetic factors and long-term development of ppFVC, while short-term intraindividual fluctuations reflect acute disease activity such as infectious exacerbations. These insights support the potential of KL-6 as a clinically relevant biomarker for CF, with applications in diagnosis, monitoring disease progression, and guiding personalized therapeutic strategies.

## Figures and Tables

**Table 1:** Clinical characteristics of pwCF at baseline of the entire cohort and separated dependent on the *MUC1* genotype of rs4072037.

	Total (*n* = 174)	*MUC1* Genotypes of rs4072037 (*n* = 158)		
		A/A (*n* = 115)	A/G (*n* = 5)	G/G (*n* = 38)
**Age**, years [mean (SD)]	32.3 (11.6)	32 (11.6)	34 (11.6)	34 (12.1)
**Gender**, ref. male, *n* (%)	102 (58.6)	70 (60.9)	2 (40.0)	24 (63.2)
**Body mass index**, kg/m^2^	20.7 (3.4)	21.5 (3.5)	18.6 (2.0)	20.5 (3.5)
*F508del* mutation, *n* (%)				
Homozygous	77 (44.3)	49 (42.6)	3 (60.0)	17 (44.7)
Compound heterozygous	54 (31.0)	36 (31.3)	2 (40.0)	12 (31.6)
Other	43 (24.7)	30 (26.1)	0 (0.0)	9 (23,7)
**Sweat chloride concentration test** mean (SD), mmol/L	93.0 (25.1)	88.4 (24.1)	78.0 (48.5)	95.3 (23.6)
**ppFEV_1_**	56.7 (24.6)	59.1 (25.0)	49.1 (22.5)	37.6 (13.7)
**ppFVC**	76.1 (21.5)	78.0 (21.6)	54.8 (14.5)	71.2 (21.3)
**Pancreatic insufficiency**, *n* (%)				
Yes	156 (89.7)	101 (87.8)	5 (100.0)	34 (89.5)
No	18 (10.3)	14 (12.2)	-	4 (10.5)
**Calprotectin**, ng/mL	284.6 (480.3)	257.0 (453.9)	21.0 (29.7)	442.8 (600.0)
**CF-related diabetes**, *n* (%)	78 (44.8)	48 (41.7)	3 (60.0)	18 (47.4)
Mean HbA1c, %	5.5 (2.3)	5.5 (2.4)	5.5 (3.2)	5.7 (1.7)
**C-reactive protein**, mg/dL	1.8 (2.8)	1.61 (2.5)	2.7 (2.5)	2.3 (3.8)
**Blood leukocytes**, /nL	9.9 (3.5)	10.0 (3.5)	8.4 (5.4)	10.2 (3.2)
**Serum IgG**, g/L	15.0 (4.8)	14.8 (4.5)	21.5 (5.0)	15.4 (4.8)
**Infectious exacerbation**, *n* (%)				
Yes	28 (16.1)	16 (13.9)	2 (40.0)	9 (23.7)
No	146 (83.9)	99 (86.1)	3 (60.0)	29 (76.3)
**Microbial colonization in sputum**, *n* (%)				
Pseudomonas aeruginosa	34 (19.5)	20 (17.4)	2 (40.0)	11 (28.9)
Staphylococcus aureus	18 (10.3)	12 (10.4)	0 (0.0)	2 (5.3)
Aspergillus fumigatus	5 (2.9)	4 (3.5)	0 (0.0)	0 (0.0)
Mycobacterium abscessus	1 (0.6)	0 (0.0)	0 (0.0)	1 (2.6)
Other microbial colonization	8 (4.6)	6 (5.2)	0 (0.0)	2 (5.3)
Colonization with multiple organisms	83 (47.7)	55 (47.8)	3 (60.0)	19 (50.0)
No colonization	24 (13.8)	17 (14.8)	0 (0.0)	3 (7.9)

Values are expressed as mean (SD) unless otherwise indicated. IgG, immunoglobulin G; ppFEV_1_, percent predicted forced expiratory volume in 1 s; ppFVC, percent predicted forced vital capacity. CF, cystic fibrosis; HbA1c, glycosylated hemoglobin.

**Table 2:** Genotype distribution, minor allele frequency (MAF), and Hardy–Weinberg equilibrium (HWE) of rs4072037 in healthy controls and pwCF.

Cohort	N	A/A	A/G	G/G	MAF	HWE *p*-Value
Healthy controls	30	9	12	9	0.50	0.27
pwCF	158	115	5	38	0.26	<0.01
Total	188	124	17	47	0.32	<0.01

**Table 3:** KL-6 concentrations dependent on rs4072037 at baseline. Pairwise comparisons were performed using Dunn–Bonferroni post hoc tests. Dominant model compares AA vs. A/G + G/G; recessive model compares AA + A/G vs. G/G.

Group	A/A	A/G	G/G	*p*-Values			
				Between-Group Comparison ^a^	Pairwise Comparisons ^b^	Dominant *p* (AA vs. A/G + G/G) ^a^	Recessive *p* (AA + A/G vs. G/G) ^a^
pwCF (*n* = 158)	396.94 ± 147.52	522.65 ± 163.51	672.16 ± 672.53	<0.001	A/A vs. A/G: 0.302 A/A vs. G/G: 0.000015 A/G vs. G/G: 1.000	<0.001	<0.001
Controls (*n* = 30)	250.56 ± 74.50	266.50 ± 67.69	337.22 ± 147.60	0.391	A/A vs. A/G: 0.554 A/A vs. G/G: 0.297 A/G vs. G/G: 0.277	0.331	0.197

Values are mean ± standard deviation. pwCF, people with cystic fibrosis. ^a^ Kruskal–Wallis test. ^b^ Dunn–Bonferroni test in pwCF; Kruskal–Wallis test in controls.

**Table 4:** Associations between KL-6 levels and clinical, inflammatory, and functional parameters at baseline.

	*N*	KL-6 Mean ± SD (U/mL)	Spearman ρ/Effect Size	*p*-Value
**Body mass index**, kg/m^2^	169	–	−0.304	<0.001
**Infectious exacerbation (yes vs. no)**	28/136	560.6 ± 220.7 vs. 438.4 ± 381.9	R = 0.28	<0.001
**C-reactive protein**, mg/dL	165	–	0.429	<0.001
**Blood leukocytes**, /nL	164	–	0.328	<0.001
**Serum IgG**, g/L	111	–	0.263	0.005
**ppFVC**	163	–	−0.610	<0.001
**ppFEV_1_**	163	–	−0.608	<0.001
**Age**, years	169	–	0.078	0.313
**Sex (male vs. female)**	98/71	453.0 ± 178.0 vs. 465.0 ± 511.7	–	0.056
***CFTR*** **mutation (homozygous *F508del*, compound, other)**	75/52/42	486.1 ± 490.7/445.4 ± 201.6/423.8 ± 184.7	–	0.749
**Pancreatic insufficiency (yes vs. no)**, *n* (%)	18/151	436.1 ± 194.9 vs. 460.7 ± 372.0	–	0.838
**Calprotectin**, ng/mL	13–14	–	0.413	0.161
**CF-related diabetes (yes vs. no)**	78/91	501.6 ± 480.4 vs. 420.8 ± 193.3	–	0.066
**HbA1c**, %	169–174	–	0.126	0.104
**Sweat chloride concentration test**, mmol/L	–	–	–	–

IgG, immunoglobulin G; ppFEV_1_, percent predicted forced expiratory volume in 1 s; ppFVC, percent predicted forced vital capacity. CF, cystic fibrosis; HbA1c, glycosylated hemoglobin.

**Table 5:** KL-6 correlations (Spearman-Rho, 95% CI, Fisher Z) for clinical parameters and group comparisons (mean ± SD, effect size R) by *MUC1* genotype at baseline. Fisher Z comparisons were not calculated for groups with very small sample sizes (*n* ≤ 5) due to unreliable estimates.

	*MUC1* Genotype	Spearman ρ/Mean ± SD	95% CI/Effect	Fisher Z Comparisons (Z_diff, *p*)
**Body mass index**, kg/m^2^	A/A *(n* = 112)	−0.313	−0.473 to −0.134	A/A vs. G/G: −0.20, 0.841 A/A vs. A/G: 0.52, 0.605 G/G vs. A/G: 0.56, 0.574
	G/G (*n* = 37)	−0.277	−0.552 to 0.052	
	A/G (*n* = 5)	−0.600	−0.969 to 0.600	
**C-reactive protein**, mg/dL	A/A (*n* = 112)	0.409	0.240 to 0.554	A/A vs. G/G: −0.74, 0.458 A/A vs. A/G: 0.02, 0.988 G/G vs. A/G: 0.22, 0.829
	G/G (*n* = 37)	0.523	0.240 to 0.724	
	A/G (*n* = 5)	0.400	−0.745 to 0.948	
**Blood leukocytes**, /nl	A/A (*n* = 112)	0.270	0.085 to 0.448	A/A vs. G/G: −0.297, 0.766 A/A vs. A/G: −0.364, 0.716 G/G vs. A/G: −0.067, 0.947
	G/G (*n* = 37)	0.519	0.260 to 0.724	
	A/G (*n* = 5)	0.564	−0.611 to 0.947	
**Serum IgG**, g/L	A/A (*n* = 112)	0.304	0.090 to 0.500	A/A vs. G/G: 0.352, 0.725 A/A vs. A/G: - G/G vs. A/G: -
	G/G (*n* = 37)	−0.038	−0.392 to 0.327	
	A/G (*n* = 3)	−1.000	−1.000 to 1.000	
**ppFVC**	A/A (*n* = 112)	−0.635	−0.732 to −0.502	A/A vs. G/G: 0.217, 0.828 A/A vs. A/G: −0.446, 0.656 G/G vs. A/G: −0.663, 0.507
	G/G (*n* = 37)	−0.750	−0.882 to −0.548	
	A/G (*n* = 5)	−0.300	−0.941 to 0.708	
**ppFEV_1_**	A/A (*n* = 112)	−0.591	−0.713 to −0.455	A/A vs. G/G: 0.227, 0.820 A/A vs. A/G: 0.015, 0.988 G/G vs. A/G: −0.212, 0.832
	G/G (*n* = 37)	−0.718	−0.857 to −0.531	
	A/G (*n* = 5)	−0.600	−0.941 to 0.708	
**Infectious exacerbation**	A/A (*n* = 112)	65.94 ± 0.457 vs. 53.12 ± 0.457	R = −0.14, ns	-
	G/G (*n* = 37)	26.44 ± 0.515 vs. 15.85 ± 0.515	R = −0.44, *p* = 0.009	-
	A/G (*n* = 5)	3.50 ± 0.837 vs. 1.50 ± 0.837	R = −0.77, ns	-

ns, not significant; IgG, immunoglobulin G; ppFEV_1_, percent predicted forced expiratory volume in 1 s; ppFVC, percent predicted forced vital capacity.

**Table 6:** Longitudinal mixed-effects model of KL-6 concentration: inter- and intraindividual effects of clinical and genetic predictors.

	Interindividual Effect		Intraindividual Effect	
Predictor	F (df1, df2)	*p*-Value	F (df1, df2)	*p*-Value
**MUC1 genotype**	F(2, 216) = 19.77	<0.001	—	—
**Infectious exacerbation**	F(2, 216) = 3.02	0.051	F(2, 216) = 0.50	0.607
**Body mass index**	F(1, 216) = 1.12	0.291	F(1, 216) = 1.10	0.295
**C-reactive protein**	F(1, 216) = 1.28	0.260	F(1, 216) = 0.08	0.772
**Blood leukocytes**	F(1, 216) = 1.31	0.254	F(1, 216) = 0.55	0.458
**Serum IgG**	F(1, 216) = 3.28	0.071	F(1, 216) = 0.06	0.803
**ppFVC**	F(1, 216) = 15.79	<0.001	F(1, 216) = 0.01	0.938
**ppFEV_1_**	F(1, 216) = 2.07	0.152	F(1, 216) = 0.25	0.616

IgG, immunoglobulin G; ppFEV_1_, percent predicted forced expiratory volume in 1 s; ppFVC, percent predicted forced vital capacity.

**Table 7:** Model-based regression analysis of intraindividual KL-6 concentration over time.

Predictor	Intraindividual Comparison	
	F (df1, df2)	*p*-Value
**Days since baseline**	F(1, 131) = 0.559	0.456
**Infection exacerbation**	F(3, 131) = 3361.10	<0.001
**Body mass index**	F(1, 131) = 1.797	0.182
**C-reactive protein**	F(1, 131) = 0.567	0.453
**Blood leukocytes**	F(1, 131) = 1.254	0.265
**Serum IgG**	F(1, 131) = 0.091	0.764
**ppFVC**	F(1, 131) = 0.057	0.812
**ppFEV_1_**	F(1, 131) = 0.019	0.892

IgG, immunoglobulin G; ppFEV_1_, percent predicted forced expiratory volume in 1 s; ppFVC, percent predicted forced vital capacity.

## Data Availability

The data analyzed in this study are not publicly available. The data presented in this study are available on request from the corresponding author due to privacy, legal and ethical reasons.

## Abbreviations

The following abbreviations are used in this manuscript:

BMI	Body mass index
CF	Cystic fibrosis
CFRD	CF-related diabetes mellitus
CFTR	Cystic fibrosis transmembrane conductance receptor
CRP	C-reactive protein
EMMs	Estimated marginal means
FEV_1_	Forced expiratory volume in 1 s
HbA1c	Glycosylated hemoglobin
IgG	Immunoglobulin G
KL-6	Krebs von den Lungen-6
LDH	Lactat dehyrodgenase
LMMs	Linear mixed-effects models
MAFs	Minor allele frequencies
*MUC1*	Mucin-1
ppFEV_1_	Percent predicted forced expiratory volume in 1 s
ppFVC	Percent predicted forced vital capacity
pwCF	People with cystic fibrosis
SARS-CoV-2	Severe acute respiratory syndrome coronavirus 2
SNP	Single nucleotide polymorphism
WBE	West German Biobank Essen
